# Identification of the role of MED6 in the development and prognosis of lung adenocarcinoma based on multi-omics profiling

**DOI:** 10.7150/jca.110981

**Published:** 2025-04-13

**Authors:** Changqing Yang, Ding Cheng, Shuo Wang, Baichuan Wang, Yingxi Li, Guixin Wang, Xingkai Wang, Cangchang Shi, Yao Tian, Keyun Zhu, Jing Feng

**Affiliations:** 1 Department of Respiratory and Critical Care Medicine, Tianjin Medical University General Hospital, Tianjin, 300052, China.; 2 Department of Plastic Surgery and Medical Aesthetics, The Second Hospital of Tianjin Medical University, Tianjin, 300211, China.; 3 The First Department of Breast Cancer, Key Laboratory of Cancer Prevention and Therapy, Tianjin's Clinical Research Center for Cancer, National Clinical Research Center for Cancer, Key Laboratory of Breast Cancer Prevention and Therapy, Tianjin Medical University Cancer Institute and Hospital, Tianjin Medical University, Tianjin, 300060, China.; 4 Anhui Chest Hospital, Hefei, Anhui Province, 23002, China.; 5 Immunology Department, Key Laboratory of Immune Microenvironment and Disease (Ministry of Education), Tianjin Medical University, Tianjin, 300070, China.; 6 Department of General Surgery, Tianjin Medical University General Hospital, Tianjin Key Laboratory of Precise Vascular Reconstruction and Organ Function Repair, Tianjin General Surgery Institute, Tianjin, 300052, China.; 7 Department of Thoracic Surgery, Ningbo Medical Center Lihuili Hospital, Ningbo University, Ningbo, Zhejiang Province, 315040, China.

**Keywords:** lung adenocarcinoma, Mediator subunits, prognosis, multi-omics, tumor microenvironment, treatment

## Abstract

**Background:** Lung adenocarcinoma (LUAD) is the most common subtype of lung cancer. Recent studies have highlighted the importance of Mediator complex subunits in cancer, but their specific roles in LUAD are still unclear.

**Methods:** The CRISPR-Cas9 loss-of-function data was used to assess gene dependency in cell growth. RNA-seq data were analyzed to evaluate the prognostic value of Mediator subunits and explore their downstream pathways. Single-cell sequencing data were utilized to examine the tumor microenvironment in LUAD. A drug sensitivity analysis was performed to identify potential therapeutic options.

**Results:** Mediator complex subunit 6 (MED6) was found to influence tumor cell growth in LUAD. Additionally, MED6 expression levels were associated with patient prognosis. MED6-positive tumor cells showed more active interactions with other cells in the LUAD microenvironment, promoting tumor progression. Based on MED6 expression, drugs such as paclitaxel, afatinib, and brivanib were identified as potential treatments.

**Conclusions:** This study revealed the role of MED6 in LUAD and its potential as a biomarker. Our findings suggest that MED6 has an effect on LUAD progression and provide valuable insights for patient stratification and personalized treatment strategies.

## Introduction

Lung cancer is the most prevalent and lethal malignancy globally, with the highest incidence and mortality rates 1. Among its subtypes, lung adenocarcinoma (LUAD) continues to show rising incidence, yet the overall prognosis for LUAD patients remains poor 2-4. In recent years, multi-omics technologies have provided critical insights into gene regulation, epigenetic changes, and the tumor microenvironment in LUAD pathogenesis and progression 5-8. While advances in treatment strategies have improved outcomes for some patients 9, tumor heterogeneity remains a significant challenge, leading to differences in disease progression and drug resistance 10-12. As such, personalized identification, management, and treatment of LUAD patients remain urgent issues that need to be addressed.

Gene transcription is a fundamental biological process. As part of the preinitiation complex (PIC), RNA polymerase II (Pol II) catalyzes DNA transcription to synthesize mRNA, snRNA, and precursor microRNA 13, 14. The Mediator complex, consisting of the head, middle, tail, and CDK8 kinase modules, plays a crucial role in transcription regulation 15, 16. In collaboration with transcription factors, Mediator helps Pol II interact with enhancer-promoter loops to control gene expression 17-19. Recent studies have highlighted the Mediator's involvement in diseases, including viral infections and cancer 20-22. Given the separation between Pol II and the Mediator, variations in Mediator subunit expression may impact the formation of Mediator-mediated condensates in tumors 23-25. Several studies have shown that Mediator subunits can promote tumorigenesis through various mechanisms 26-28, suggesting that they may have diverse roles in cancer and warrant further exploration.

In our study, we identified MED6 through tumor cell proliferation assays and differential expression analysis in LUAD. By integrating single-cell sequencing and RNA-seq data, we found that MED6 may interact with other cells in the tumor microenvironment. Based on MED6 expression, we also predicted potential therapeutic drugs. These results suggest that MED6 plays a key role in LUAD development and progression, highlighting its potential as both a biomarker and a therapeutic target.

## Materials and Methods

### Public and online data collection

The CRISPR-Cas9 loss-of-function data were obtained from the Dependency Map (DepMap) portal (https://depmap.org/portal/) 29. A total of 49 LUAD cell lines from DepMap were selected for analysis. RNA-seq data for LUAD were retrieved from The Cancer Genome Atlas Program (TCGA) via the Xena platform 30 (https://xena.ucsc.edu/). Single-cell sequencing data were sourced from a published study 31 and downloaded from the Code Ocean capsule database (10.24433/CO.0121060.v1). Survival analysis of specific genes was performed using GEPIA2 32 (http://gepia2.cancer-pku.cn/#index). All local data analyses were conducted using R software (version 4.4.1).

### Identification of essential MED family genes

The CERES score, included in the CRISPR-Cas9 loss-of-function data, was used to estimate gene dependency for cell growth, following the method outlined in a previous study 33. A lower CERES score indicates greater gene dependence for cell growth. The proportion of cell lines with CERES scores below -1 was used to determine gene dependency, with a threshold of >80%. Genes with a mean CERES score below -1 and a proportion >80% were classified as essential for cell growth. MED family genes were retrieved from GeneCards 34 (https://www.genecards.org/).

### Differential genes and enrichment analysis

The RNA-seq data in the form of transcripts per million (TPM) were standardized using log2-transformed to obtain gene expression data for analysis. MED6 high- and low-expression groups were stratified based on the median MED6 expression level in the TCGA LUAD cohort. Differentially expressed genes (DEGs) were identified using the limma package (version 3.60.4) 35, comparing LUAD versus normal tissue and high versus low MED6 groups. DEGs were selected based on an adjusted *P*-value of <0.05 and a log2 fold-change (log2FC) > 0. The top 500 DEGs meeting these criteria were used for gene enrichment analysis using the clusterProfiler package (version 4.10.1) 36, based on the biological process from Gene Ontology (GO) 37, 38 and the Kyoto Encyclopedia of Genes and Genomes (KEGG) 39, 40. Gene Set Enrichment Analysis (GSEA) was performed using the hallmark gene sets 41.

### Single-cell data processing and analysis

Single-cell processing followed the official Seurat R package workflow (version 4.4.0) 42. Low-quality cells were removed based on the following criteria: total counts < 500, gene numbers < 200, and mitochondrial gene percentage > 20%. Batch effects and doublets were removed using Harmony (version 1.2.0) 43 and DoubletFinder (version 2.0.3) 44. Gene expression normalization was performed using SCTransform command. The resolution of cell clustering was evaluated by clustree (version 0.5.1) and a proper resolution of 0.5 was set to obtain different cell clusters. Cell types were annotated based on marker gene expression, as described in previous studies 31, 45. Copy number variation (CNV) analysis was conducted to identify malignant cells, using the infercnv package (version 1.14.2). According to previous studies 45, 46, malignant cells were defined by a CNV score > 0.001 and CNV correlation > 0.4. MED6-positive and -negative cells were separated based on a gene expression threshold of 0. Cell communication was analyzed using the CellChat package (version 1.6.1), with default parameters and the human ligand-receptor database 47.

### Drug sensitivity and screening analysis

Drug sensitivity analysis was performed using the Genomics of Drug Sensitivity in Cancer (GDSC) public database 48. The oncoPredict package (version 1.2) was used to download associated data from GDSC 49. This package constructs a regression model for gene expression and drug sensitivity based on the known GDSC gene expression profiles of cell lines and their sensitivity to various drugs. Drug sensitivity scores were imputed based on the IC50 values for each drug in GDSC. Thus, drug sensitivity could be predicted in the MED6 high and low groups. Intergroup differences were assessed using a t-test, with *P*-values < 0.05 considered statistically significant.

## Results

### Screening the cell growth required genes in LUAD cell lines

**Figure 1:**
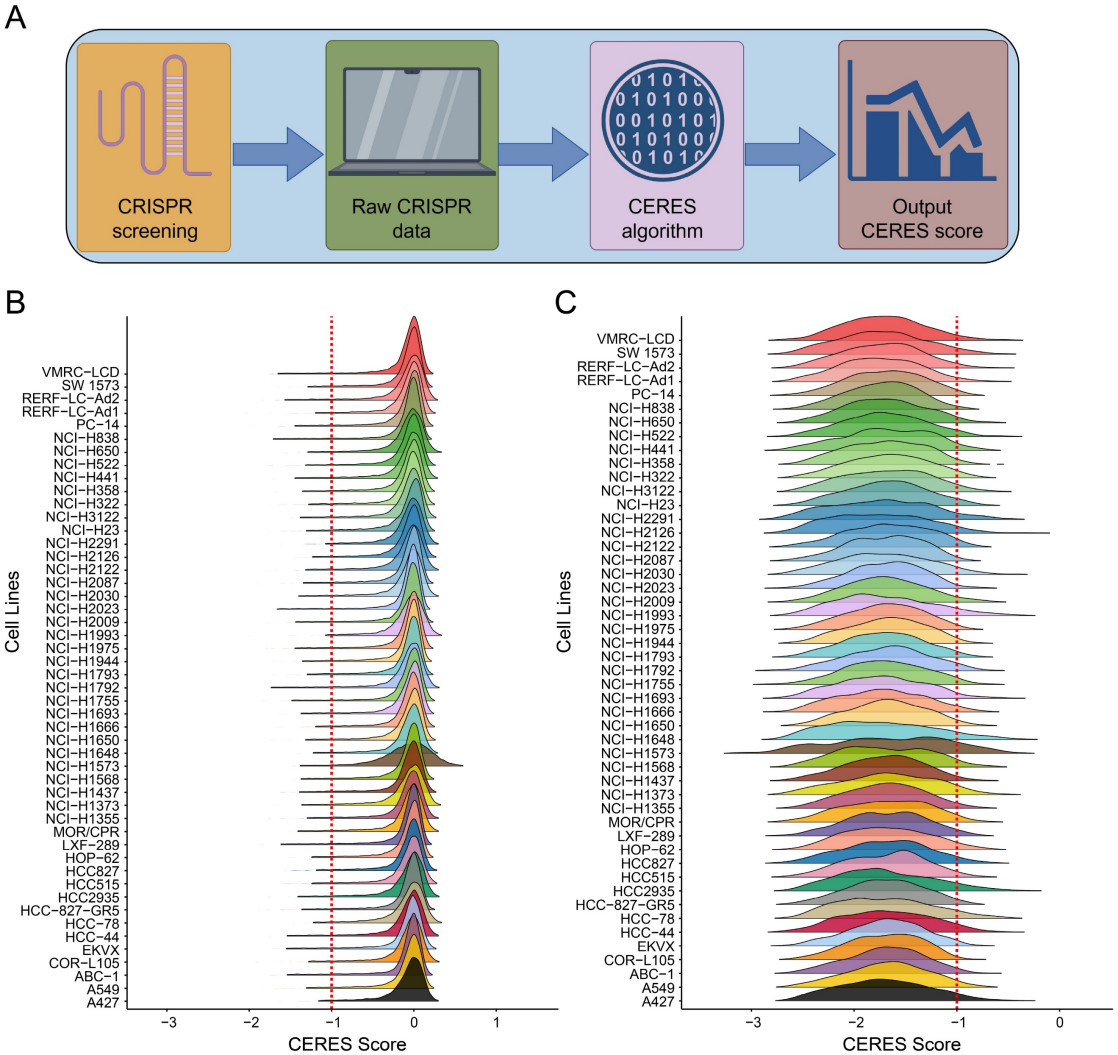
** CRISPR-Cas9 Screening in LUAD Cell Lines.** (**A**) Workflow of the CRISPR-Cas9 screening process. (**B**) Distribution of overall CERES scores for all genes across LUAD cell lines. (**C**) Distribution of CERES scores for genes with scores below -1 in more than 80% of LUAD cell lines.

The workflow for screening cell growth-required genes is shown in Figure 1A. The CRISPR-Cas9 system was used for selective knockout of each gene to calculate CERES scores. The CERES scores of genes were predominantly distributed around zero across different LUAD cell lines (Figure 1B), indicating that most genes are not necessarily involved in tumor cell growth in LUAD. To identify genes essential for tumor cell growth, we focused on genes with CERES scores lower than -1 in over 80% of the cell lines. Genes meeting this criterion showed a concentrated distribution between -2 and -1 for their CERES scores (Figure 1C). As a result, 659 genes were identified as essential for tumor cell growth in LUAD (Table S1).

### MED6 was upregulated in LUAD with prognostic value

**Figure 2:**
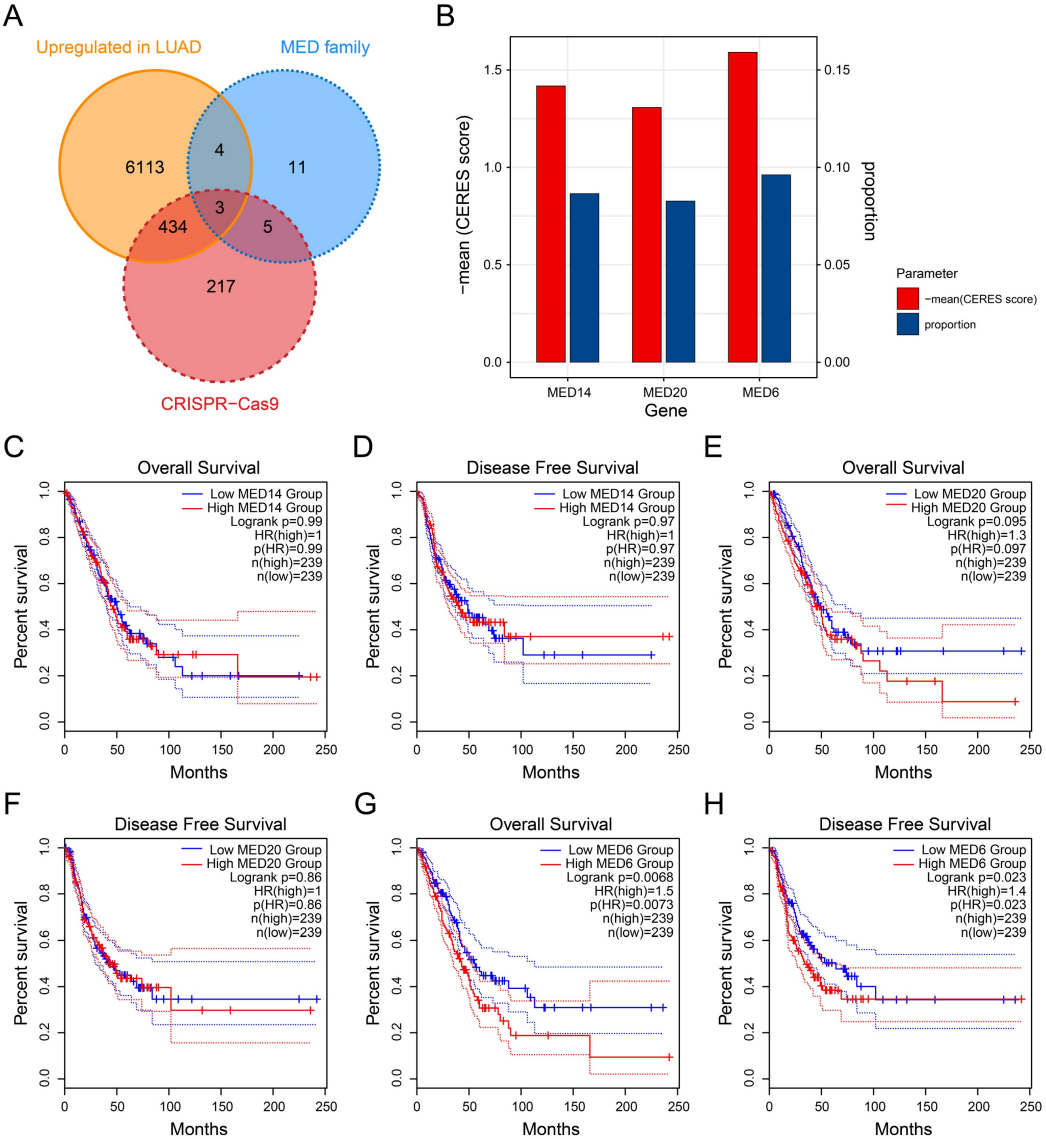
** Identification of MED6 as a Prognostic Marker.** (**A**) CRISPR-Cas9 screening of key MED family genes upregulated in LUAD. (**B**) Mean CERES scores and proportion parameters of MED family genes. (**C-H**) Kaplan-Meier survival analysis for high/low expression groups of MED14 (**C-D**), MED20 (**E-F**), and MED6 (**G-H**) with respect to overall survival (OS) (**C, E, G**) and disease-free survival (DFS) (**D, F, H**).

To assess whether the MED family genes are essential for LUAD, differential expression analysis was performed between LUAD and normal tissue, focusing on the MED gene family. Among them, only three genes—MED6, MED14, and MED20—were found to be highly expressed in LUAD and essential for tumor cell growth (Figure 2A). MED6 exhibited the lowest CERES score and the highest dependency for cell growth, indicating that it is more critical for tumor proliferation than the other genes (Figure 2B). Survival analysis revealed that MED14 and MED20 did not show significant prognostic value for overall survival (OS) or disease-free survival (DFS) in LUAD patients (Figure 2C-F). However, MED6 emerged as a significant prognostic marker for both OS and DFS (Figure 2G-H). These findings suggest that high MED6 expression is associated with poor prognosis in LUAD.

### Possible cellular pathways affected by MED6

**Figure 3:**
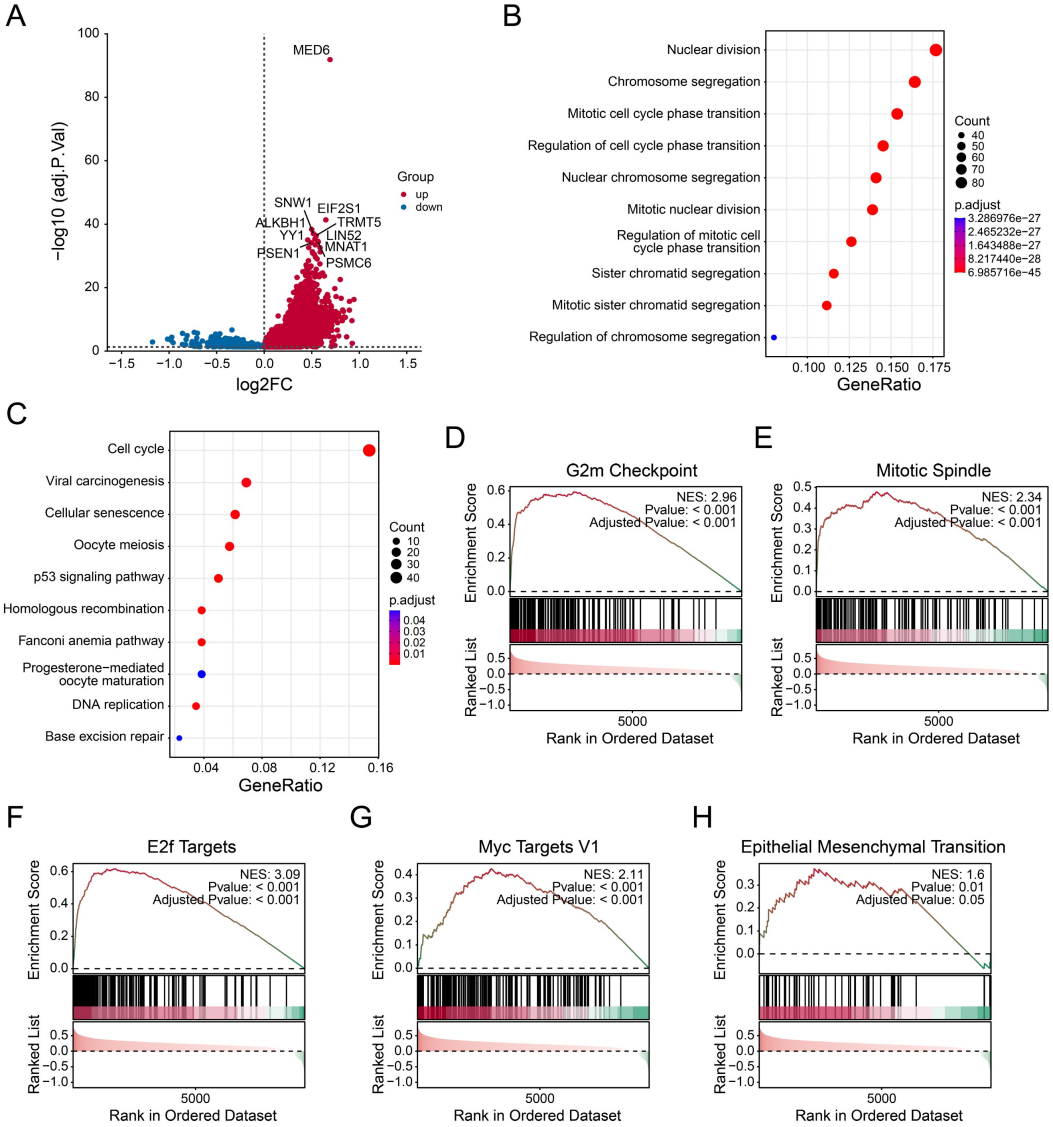
** Potential Pathways Affected by MED6.** (**A**) Differentially expressed genes (DEGs) between high and low MED6 groups. (**B**) GO enrichment analysis of DEGs. (**C**) KEGG pathway enrichment analysis of DEGs (**D-H**) GSEA revealing significant pathways in the high versus low MED6 groups, (**D**) G2/M checkpoint, (**E**) Mitotic spindle, (**F**) E2F targets, (**G**) Myc targets V1, (**H**) Epithelial-mesenchymal transition. NES corresponds to normalized enrichment score.

To explore the downstream functions of MED6, differential expression analysis was performed by stratifying the samples into MED6-high and MED6-low groups. The majority of DEGs were upregulated in the MED6-high group, including SNW1, EIF2S1, and ALKBH1 (Figure 3A). These results suggested that MED6 regulates numerous genes, and their expression changes correlate with MED6 expression in LUAD. The DEGs that exhibited expression patterns similar to MED6 were primarily involved in processes such as nuclear division, chromosome segregation, the cell cycle, DNA replication, and homologous recombination (Figure 3B-C). GSEA revealed significant enrichment of cell cycle-related pathways in the MED6-high group (Figure 3D-I). Additionally, the epithelial-mesenchymal transition (EMT) pathway was upregulated in the MED6-high group, suggesting that MED6 may enhance tumor cell motility and migration (Figure 3J). These findings indicate that MED6 may promote tumor growth and progression in LUAD.

### Identification of MED6 in malignant cells

**Figure 4:**
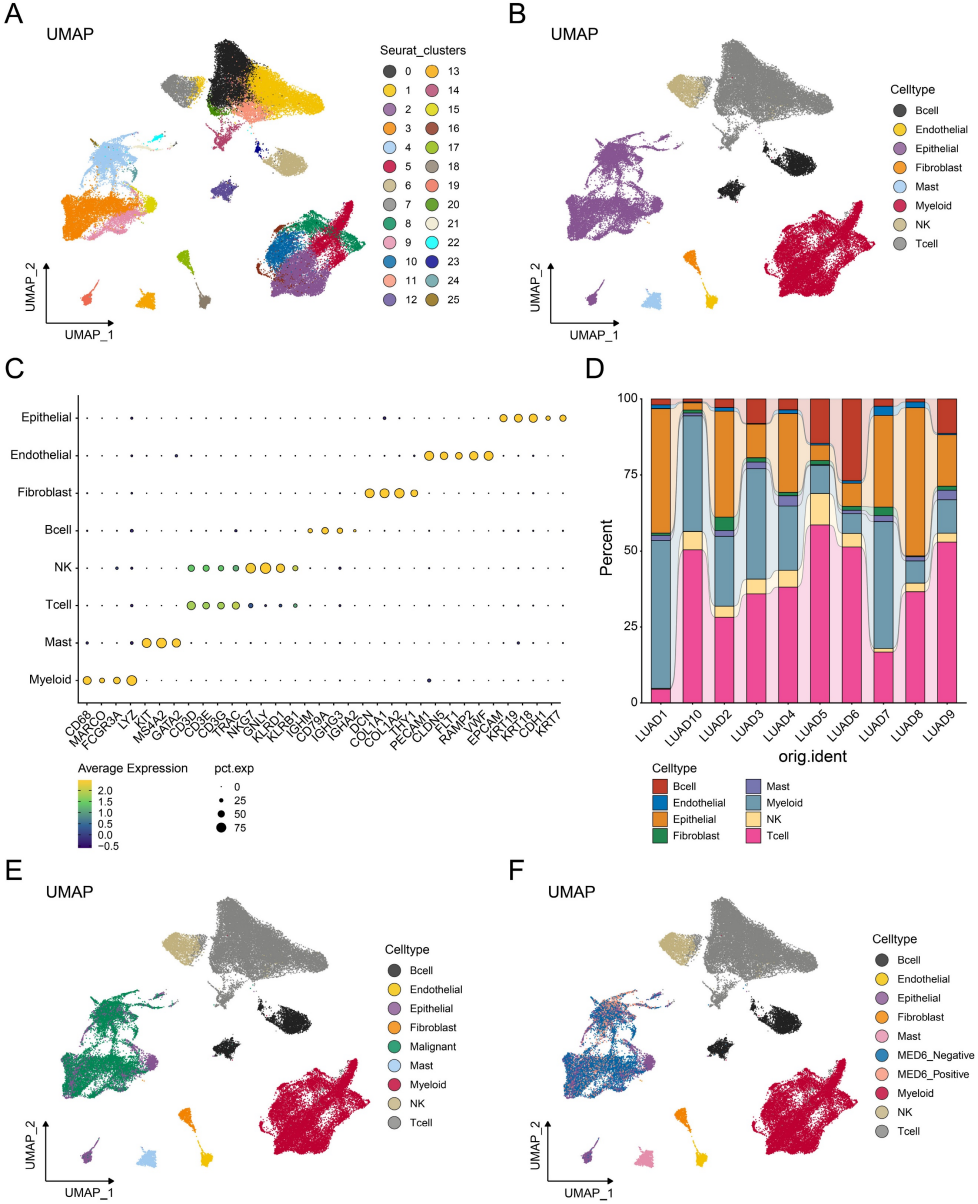
** Single-Cell Transcriptome Analysis of LUAD.** (**A**) Uniform Manifold Approximation and Projection (UMAP) showing different cell clusters. (**B**) UMAP depicting different cell types. (**C**) Annotation markers for each identified cell type. (**D**) Proportions of each cell type across different samples. (**E**) UMAP of various cell types, including malignant cells. (**F**) UMAP of cell types, highlighting MED6-positive and MED6-negative cells.

Single-cell analysis was performed to investigate the role of MED6 in tumor cells. A total of 58,191 cells and 25 clusters were obtained after processing and removing batch effects (Figure 4A). Eight cell types were identified and clearly distinguished from each other (Figure 4B), with cell type markers consistent with previous studies 31, 45 (Figure 4C). The proportion of each cell type slightly varied across different samples (Figure 4D). However, epithelial, myeloid, and T cells were the predominant cell types in each sample. Since LUAD is primarily derived from epithelial cells 50, the CNV analysis was performed in epithelial cells. Malignant tumor cells were identified based on high CNV scores and correlations (Figure S1A-J). Consistently, these malignant cells were separated from other cell types (Figure 4E). Based on MED6 expression, tumor cells were divided into MED6-positive and MED6-negative cells, resulting in 10 distinct cell types (Figure 4F). These findings suggest that MED6 expression may provide new molecular insights into LUAD.

### Interaction between MED6 positive tumor cells and others

**Figure 5:**
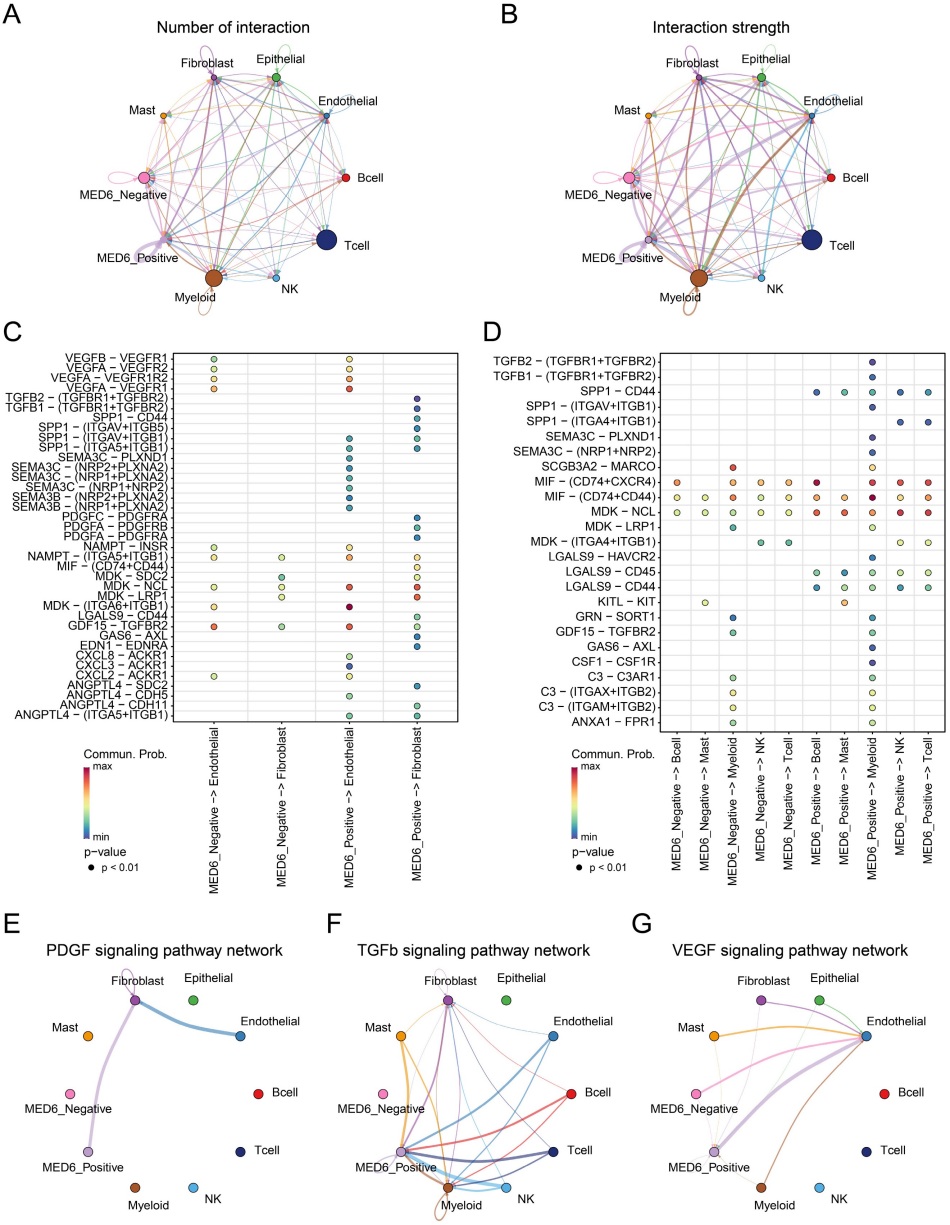
** Cell-Cell Communication Between Different Cell Types.** (**A**) Number of interactions between various cell types. (**B**) Interaction strength between different cell types. (**C**) Interaction network between MED6-related tumor cells and stromal cells. (**D**) Interaction network between MED6-related tumor cells and immune cells. (**E-G**) Specific signaling pathway networks in distinct cell types, (**E**) PDGF signaling pathway, (**F**) TGF-β signaling pathway, (**G**) VEGF signaling pathway. "Commun. Prob." refers to communication probability.

Cell-cell interactions within the tumor microenvironment (TME) are closely linked to tumor initiation and growth 51, 52. Therefore, cell communication analysis was conducted between MED6-positive, MED6-negative, and other cells. The analysis revealed that MED6-positive cells exhibited a higher number of interactions compared to MED6-negative cells, suggesting that more ligand-receptor pairs were detected in interactions involving MED6-positive cells (Figure 5A). Furthermore, the interaction strength was stronger with MED6-positive cells, indicating that MED6-positive cells play a central role in cell communication within the TME (Figure 5B). In interactions with fibroblasts and endothelial cells, MED6-positive cells were more likely to communicate through signaling pathways such as transforming growth factor (TGF)-β, vascular endothelial growth factor (VEGF), semaphorin (SEMA), and midkine (MDK) (Figure 5C). Additionally, MED6-positive cells exhibited stronger interactions with immune cells, particularly through the SPP1 signaling pathway (Figure 5D). The platelet-derived growth factor (PDGF) and TGFβ pathways were exclusively present in MED6-positive cells (Figure 5E-F), and MED6-positive cells showed higher intensity in the VEGF pathway (Figure 5G). These results suggest that MED6-positive tumor cells have significant interactions with other cells in the TME.

### Potential drugs were screened based on MED6

**Figure 6:**
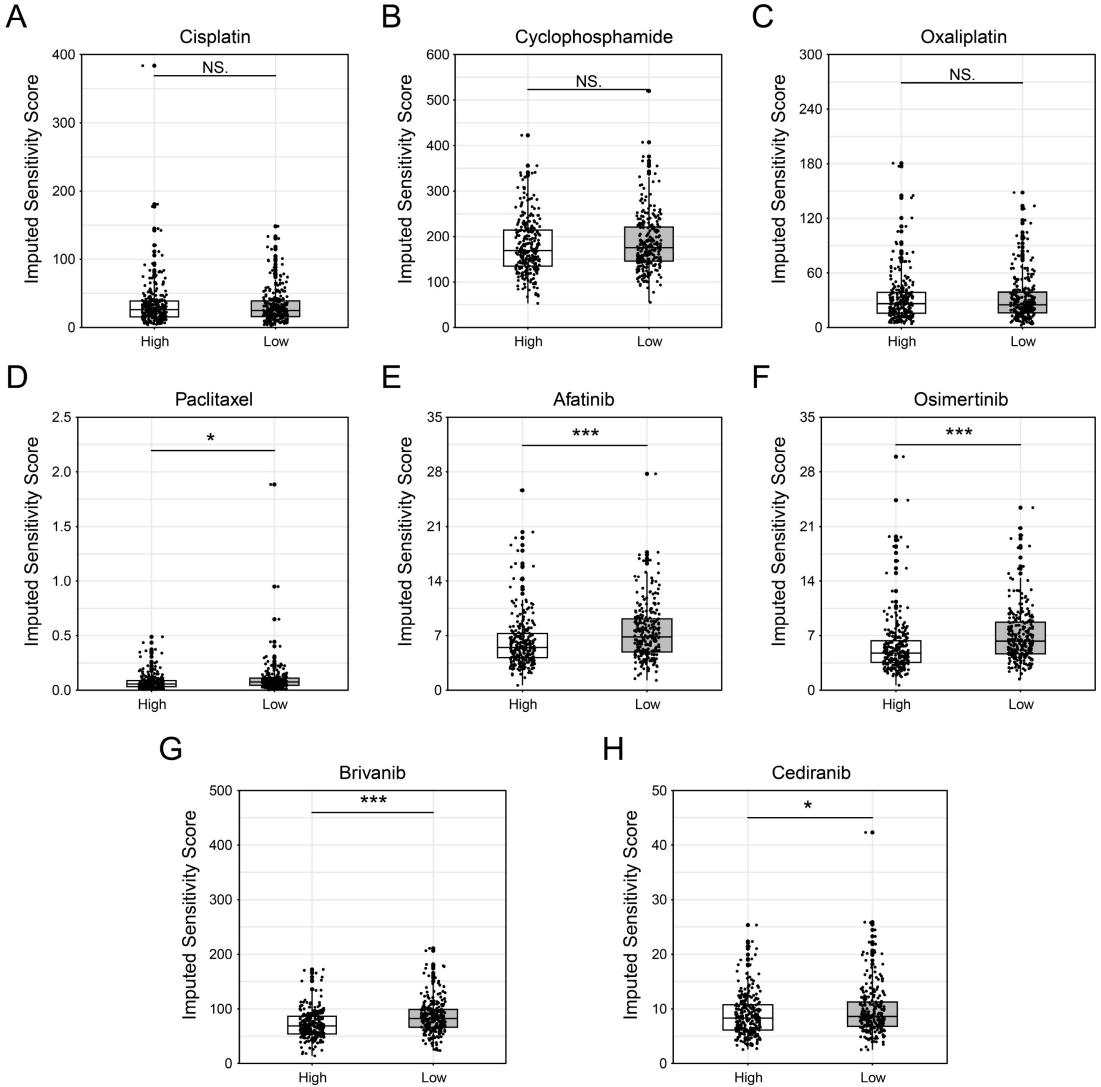
** Drug Screening Between High and Low MED6 Expression Groups.** (**A-H**) Predicted sensitivity scores for various drugs across high and low MED6 groups, (**A**) Cisplatin, (**B**) Cyclophosphamide, (**C**) Oxaliplatin, (**D**) Paclitaxel, (**E**) Afatinib, (**F**) Osimertinib, (**G**) Brivanib, (**H**) Cediranib. * *P* < 0.05, *** *P* < 0.001, NS indicates no significance.

Given that MED6 is a cell growth-dependent and prognostic gene in LUAD, potential drugs were screened for patients with high MED6 expression. Drug sensitivity was assessed using compounds from GDSC database, with IC50 values used to represent drug sensitivity in the MED6 high and low groups. Chemotherapeutic drugs such as cisplatin, cyclophosphamide, and oxaliplatin showed no significant differences between the MED6 high and low groups (Figure 6A-C). However, paclitaxel demonstrated a lower IC50 in the MED6-high group, suggesting that patients with high MED6 expression may be more sensitive to paclitaxel (Figure 6D). Additionally, the IC50 values for afatinib and osimertinib, which inhibit epidermal growth factor receptor (EGFR), were significantly lower in the MED6-high group (Figure 6E-F). Furthermore, the VEGFR and PDGFR dual inhibitors brivanib and cediranib also showed lower IC50 values in the MED6-high group (Figure 6G-H). These findings identify several drugs that may be potentially effective for LUAD patients with high MED6 expression.

## Discussion

The MED gene family encodes several Mediator subunits, which are essential for Pol II transcription 16, 53. These subunits come together to form the Mediator complex, a crucial transcriptional regulator that bridges transcription factors with other components of the pre-initiation complex (PIC), including Pol II 54. While some studies suggest that MED subunits might have oncogenic potential, the specific role of MED6 in tumors remains underexplored 55, 56. To the best of our knowledge, this is the first study to investigate the role of MED6 in lung cancer and propose its potential as a prognostic marker in LUAD.

To identify genes in the MED family, we used the CRISPR-Cas9 system to knockout genes in 49 LUAD cell lines from public databases. In over 80% of these lines, MED6 knockout significantly inhibited tumor cell growth, indicating that MED6 is closely linked to tumor cell proliferation and growth. Moreover, MED6 expression correlated with both OS and DFS in LUAD patients, suggesting its potential as a prognostic marker. This effect likely stems from the role of MED6 within the Mediator complex, which is involved in transcription. MED6, located at the head of Mediator 57, 58, coordinates PIC assembly 59, and interactions between Mediator and transcription initiation factors stabilize the complex 60. Previous studies have shown that MED6 interacts with the C-terminal domain of Pol II, a critical step in transcription initiation 61. Thus, Mediator serves as a key link, transmitting transcription factor signals to Pol II to start transcription 62. Interestingly, Mediator may also be involved in transcription-coupled export (TREX2), affecting the nuclear pore complex 63, which could influence mRNA export and further broaden Mediator's regulatory role in transcription 64. Additionally, other Mediator subunits have been linked to tumor pathology and prognosis 65, 66, reinforcing the potential of MED6 as a key player in tumor biology. These findings suggest that MED6, as part of the Mediator complex, plays a crucial role in LUAD by regulating the assembly, positioning, and transcription of the pre-transcriptional complex, making it a promising prognostic biomarker.

Since MED6 is essential for transcription, it may also affect LUAD through the expression of downstream genes. Our results showed that genes like SNW1, ALKBH1, and EIF2S1 exhibited expression patterns similar to MED6. In neuroblastoma, SNW1, a molecular chaperone, regulates the NOTCH pathway and correlates with poor prognosis 67. ALKBH1 promotes lung cancer cell migration and invasion by demethylating m6A RNA 68. EIF2S1 controls GPX4 and SLC7A11 expression to protect tumor cells from ferroptosis, thus promoting tumor growth 69. These genes may synergistically enhance LUAD progression. Additionally, we found that in MED6-high LUAD cells, the expression of cell cycle-related genes was elevated, and related pathways were significantly enriched. Consistent with previous studies, the upregulation of E2f target genes and pathways suggests independent pro-tumor activities 70, 71. Moreover, Myc-target V1 plays a critical role in tumor proliferation, migration, and angiogenesis, further promoting tumor progression 72. Abnormalities in the G2M checkpoint and mitotic spindle have been well-documented and are associated with poor prognosis in cancer patients 73, 74. Notably, the EMT pathway, which is critical for tumorigenesis and metastasis, was also enriched in the MED6-high-expression group 75, suggesting that high MED6 levels may facilitate tumor invasion and metastasis. In summary, MED6 likely promotes LUAD progression by regulating downstream gene expression and tumor cell growth.

Building on the advantages of bioinformatics technologies in cancer research 46, 76, 77, our study explored how MED6 affects interactions between tumor cells and other cells in TME at the single-cell level. Compared to MED6-negative tumor cells, MED6-positive tumor cells showed more frequent interactions with other TME cell types. Tumor-host cell interactions are crucial for tumor initiation and progression 78. Similar to previous studies, our single-cell analysis revealed diverse and complex interactions in the LUAD TME, particularly for MED6-positive tumor cells 79, 80. Secreted signals, such as ligand-receptor binding, play a major role in intercellular communication in the TME 51. For example, melanoma can induce a pro-angiogenic phenotype in bone marrow progenitor cells through exosomes, increasing metastasis 81. Additionally, CC and CXC chemokines and their receptors are expressed across various TME cell types, promoting tumor growth and immune regulation 82. Our results showed that MED6-positive tumor cells exhibited more significant activity in interactions with stromal and immune cells via secreted signals like SEMA, MDK, and SPP1. Even in pathways related to angiogenesis, invasion, metastasis, and immune suppression—such as VEGF, PDGF, and TGF-β^83^-^85^—MED6-positive tumor cells showed stronger intercellular communication. Previous research indicates that MDK signaling promotes tumor growth, metastasis, and angiogenesis 86, while SPP1 influences tumor-associated macrophages (TAMs) and immune suppression in the TME 87, 88. The SPP1-CD44 interaction impairs immune cell antigen presentation and immune responses, facilitating tumor progression 89. Although different types of SEMA have distinct roles in angiogenesis 90, 91, this highlights the complex role of MED6 in tumor biology. In conclusion, MED6 may regulate tumor cell interactions in the TME, contributing to tumor progression. Furthermore, changes in MED6 expression provide new insights into TME heterogeneity in LUAD.

Given the potential role of MED6 in LUAD tumor progression, we performed drug screening for MED6-high-expressing populations. We found no significant difference in sensitivity to cisplatin, oxaliplatin, and cyclophosphamide. As platinum-based drugs, cisplatin and oxaliplatin likely target defective DNA repair processes in tumor cells 92, while cyclophosphamide likely works by inactivating aldehyde dehydrogenase 93. These mechanisms may differ from the role of MED6 in transcription, possibly explaining the lack of significant effects in the MED6-high group. Paclitaxel, which induces mitotic arrest and apoptosis 94, aligns with the role of MED6 in promoting cell cycle activity and mitotic spindle formation. Interestingly, both brivanib and cediranib, dual inhibitors of VEGFR and PDGFR 95-98, correspond with stronger interactions between MED6-high tumor cells and stromal cells via VEGFR and PDGFR. Furthermore, crosstalk between the EGFR and VEGFR pathways promotes angiogenesis and tumor proliferation 99, 100. This suggests that EGFR inhibitors like afatinib and osimertinib may be effective for MED6-high-expression patients. In conclusion, the drugs identified in this study may offer therapeutic potential for MED6-high-expressing LUAD patients.

Although our bioinformatics analysis of MED6 provides valuable insights, there are several limitations. Further *in vivo* and *in vitro* studies are needed to validate the role of MED6 in LUAD tumor cells and its downstream effects. Additionally, the mechanisms behind MED6-positive tumor cell interactions in the TME require further investigation. Since our study samples were derived solely from TCGA, this may potentially lead to bias in the ability of MED6 to serve as a prognostic marker for LUAD patients. Future study should involve large-scale, multi-center trials to overcome the issues of small sample size and sample selection bias, in order to validate the prognostic role of MED6 and assess its robustness as a predictive biomarker. Additionally, the dataset used in this study lacks corresponding clinical information for the patients, which limits our ability to accurately evaluate the correlation and relationship between MED6 and clinical variables. Finally, the drugs identified for MED6-high-expressing patients should undergo additional testing to confirm their therapeutic efficacy and safety in clinical trials.

In conclusion, our study provides a comprehensive investigation into the role of MED6 in LUAD. Our results suggest that MED6 promotes tumor progression by influencing tumor cell proliferation and intercellular interactions within the TME, thereby impacting LUAD prognosis. Based on MED6 expression levels, more effective, personalized treatment strategies may be developed. These findings underscore MED6 as a key molecule in LUAD development, offering new insights into identifying patients with varying prognoses and exploring personalized treatment options.

## Supplementary Material

Supplementary figure and table.

## Abbreviations

LUAD: lung adenocarcinoma
MED6: Mediator complex subunit 6
PIC: preinitiation complex
Pol II: RNA polymerase II
DepMap: Dependency Map
TCGA: The Cancer Genome Atlas Program
DEGs: differentially expressed genes
log2FC: log2 fold-change
GO: Gene Ontology
KEGG: Kyoto Encyclopedia of Genes and Genomes
GSEA: Gene Set Enrichment Analysis
CNV: copy number variation
GDSC: Genomics of Drug Sensitivity in Cancer
OS: overall survival
DFS: disease-free survival
EMT: epithelial-mesenchymal transition
TME: tumor microenvironment
TGF-β: transforming growth factor-β
VEGF: vascular endothelial growth factor
SEMA: semaphorin
MDK: midkine
PDGF: platelet-derived growth factor
EGFR: epidermal growth factor receptor
TREX2: transcription-coupled export
TAMs: tumor-associated macrophages
