# Supplementary material for: Identification of the role of MED6 in the development and prognosis of lung adenocarcinoma based on multi-omics profiling
Source: J Cancer. 2025 Apr 13;16(7):2362–74. doi: 10.7150/jca.110981 (PMC12036094; doi:10.7150/jca.110981)
Supplement: Supplementary file 1 — Supplementary figure and table. [file jcav16p2362s1.pdf]

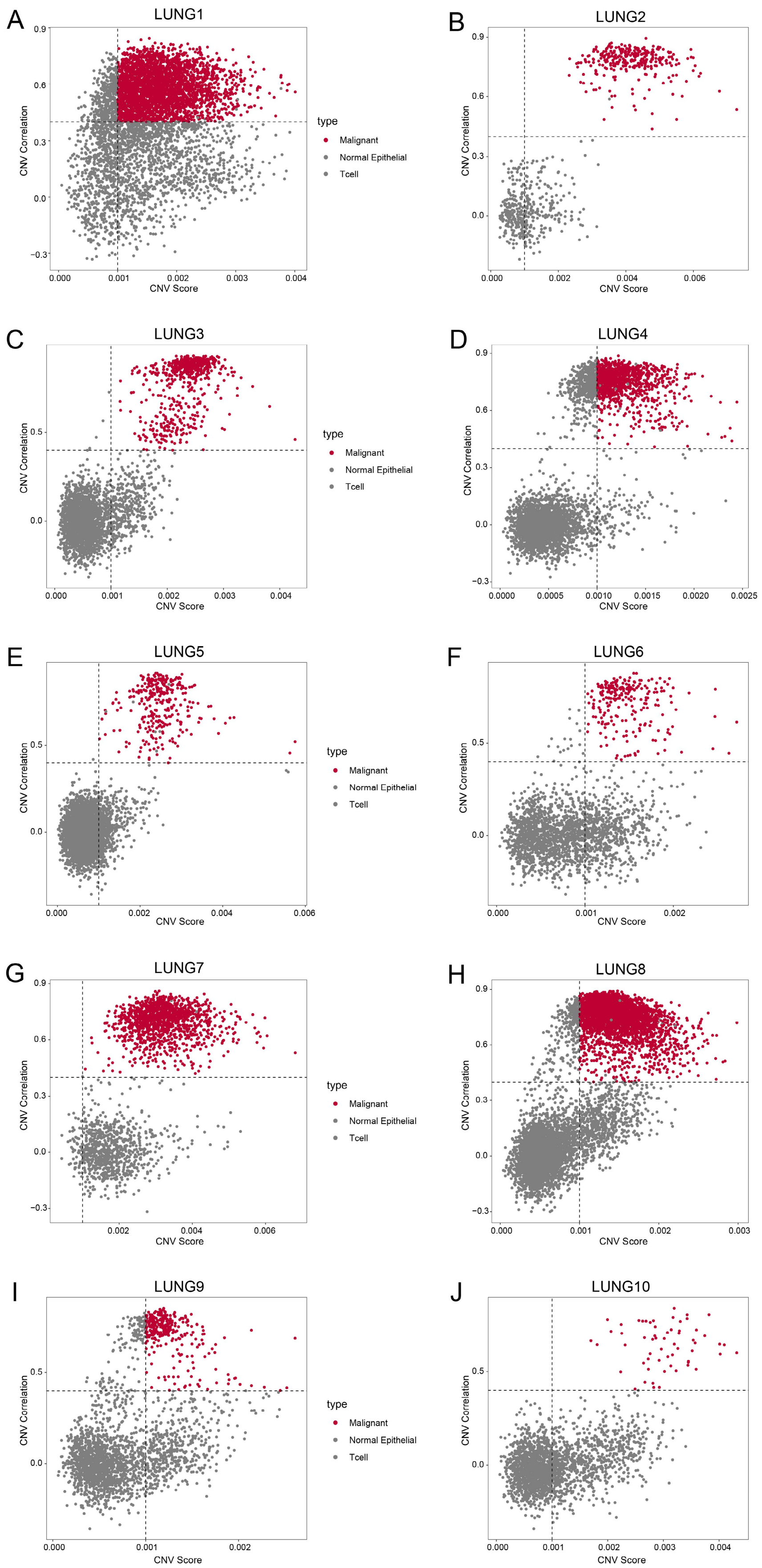
A
LUNG1
B
LUNG2
C
LUNG3
D
LUNG4
E
LUNG5
F
LUNG6
G
LUNG7
H
LUNG8
I
LUNG9
J
LUNG10
CNV Correlation
CNV Score
type
Malignant
Normal Epithelial
Tcell

1

Figure S1 CNV Analysis of Each Sample. **(A-J)** Dot plots of CNV scores and CNV correlations for T cells, normal cells, and malignant epithelial cells from **(A)** LUNG1 to **(J)** LUNG10.

Supplementary Table S1 CERES score

gene	mean	proportion
AARS1	-1.98942	0.980769
ABCE1	-1.84737	0.980769
ABCF1	-1.35112	0.980769
ACTL6A	-1.4469	0.884615
ACTR2	-1.34589	0.846154
AHCTF1	-1.37795	0.961538
AKIRIN2	-1.30347	0.923077
ALG11	-1.60637	0.942308
ALG1L	-1.27153	0.884615
ANAPC1	-1.76639	0.980769
ANAPC10	-1.30592	0.903846
ANAPC11	-2.24042	0.980769
ANAPC2	-1.78794	0.980769
ANAPC4	-2.00935	0.961538
ANAPC5	-1.65158	0.980769
ANKLE2	-2.32305	0.980769
AQR	-1.79352	0.961538
ARCN1	-1.37743	0.923077
ARIH1	-1.33951	0.826923
ARL2	-1.44775	0.903846
ATP2A2	-1.82326	0.942308
ATP5MF	-1.50786	0.884615
ATP6V0B	-1.49798	0.923077
ATP6V0C	-2.10506	0.961538
ATP6V1A	-1.81184	0.980769
ATP6V1B2	-1.77586	0.884615
ATP6V1E1	-1.68608	0.942308
ATP6V1F	-1.37605	0.826923
ATP6V1G1	-1.45395	0.903846
AURKA	-1.48349	0.923077
AURKB	-1.90221	0.980769
BANF1	-2.10346	0.980769
BANP	-1.22417	0.884615
BIRC5	-1.83043	0.980769
BRF1	-1.36751	0.903846
BRF2	-2.07702	0.980769

BUB1B	-1.31792	0.846154
BUB3	-1.93905	0.942308
BUD23	-1.70941	0.980769
BUD31	-2.20818	0.980769
BYSL	-1.47161	0.923077
C1orf131	-1.14606	0.903846
CACTIN	-1.58918	0.923077
CARS1	-1.72367	0.980769
CCNA2	-1.27168	0.865385
CCNK	-1.63894	0.961538
CCT2	-1.86833	0.961538
CCT3	-1.99707	0.980769
CCT4	-1.93194	0.980769
CCT5	-1.5896	0.980769
CCT6A	-1.66222	0.980769
CCT7	-1.70293	0.961538
CCT8	-1.50289	0.961538
CDC123	-2.18047	0.980769
CDC16	-2.13957	0.980769
CDC20	-1.95356	0.961538
CDC23	-2.1566	0.980769
CDC27	-2.51336	0.980769
CDC37	-1.36843	0.884615
CDC45	-2.30796	0.980769
CDC5L	-2.19267	0.980769
CDC6	-1.58292	0.942308
CDC7	-1.98596	0.961538
CDC73	-1.50245	0.961538
CDCA8	-1.65065	0.942308
CDK1	-2.07322	0.980769
CDK7	-1.8011	0.980769
CDK9	-1.42762	0.942308
CDT1	-1.69071	0.980769
CENATAC	-1.49414	0.942308
CENPK	-1.61709	0.942308
CENPN	-1.38602	0.923077
CENPW	-1.69195	0.923077
CFAP298	-1.87243	0.980769
CHAF1A	-1.28203	0.884615
CHAF1B	-1.86996	0.980769
CHEK1	-2.09684	0.980769
CHERP	-1.28278	0.903846

CHMP2A	-1.9246	0.980769
CHMP6	-1.70476	0.961538
CIAO2B	-1.36601	0.846154
CIAO3	-1.68455	0.980769
CKAP5	-1.65532	0.980769
CLNS1A	-1.44627	0.865385
CLP1	-1.93484	0.980769
CLTC	-1.34774	0.807692
CNIH4	-1.15587	0.865385
CNOT3	-1.57641	0.942308
COPA	-2.1405	0.980769
COPB1	-2.02218	0.980769
COPB2	-2.00328	0.961538
COPE	-1.59601	0.846154
COPS5	-1.63477	0.980769
COPS6	-1.56086	0.903846
COPZ1	-1.78845	0.961538
CPSF1	-1.28548	0.807692
CPSF2	-1.34499	0.923077
CPSF3	-1.50514	0.980769
CPSF4	-1.44202	0.923077
CRCP	-1.44631	0.865385
CRNKL1	-1.75368	0.980769
CSE1L	-2.02699	0.980769
CTCF	-1.83751	0.961538
CTDP1	-1.85921	0.980769
CWC22	-1.32694	0.826923
DAD1	-2.05119	0.980769
DARS1	-1.83263	0.980769
DBR1	-1.6677	0.961538
DDB1	-1.97852	0.980769
DDX10	-1.59646	0.961538
DDX18	-1.93411	0.980769
DDX41	-1.77518	0.961538
DDX42	-1.13207	0.807692
DDX46	-1.36077	0.980769
DDX47	-1.78816	0.980769
DDX49	-1.49223	0.961538
DDX54	-1.44486	0.961538
DDX55	-1.26812	0.807692
DDX56	-1.97259	0.980769
DHDDS	-1.87466	0.980769

DHX15	-1.89598	0.980769
DHX16	-1.60514	0.980769
DHX37	-1.58637	0.942308
DHX8	-1.56239	0.923077
DNAJC17	-1.5438	0.980769
DONSON	-2.33052	0.980769
DTL	-2.08255	0.980769
DTYMK	-1.45842	0.807692
DUT	-1.90901	0.980769
DYNC1H1	-1.94051	0.980769
DYNC1I2	-1.67778	0.961538
DYNLRB1	-1.66809	0.884615
ECD	-1.96094	0.980769
EEF1A1	-1.88557	0.980769
EEF1G	-1.48247	0.961538
EEF2	-2.30904	0.980769
EEF2KMT	-1.56941	0.980769
EFTUD2	-1.80109	0.980769
EIF1AD	-1.26329	0.826923
EIF1AX	-1.68345	0.826923
EIF2B1	-1.16664	0.807692
EIF2B2	-1.72393	0.980769
EIF2B3	-1.57592	0.923077
EIF2B4	-1.8107	0.980769
EIF2B5	-1.44822	0.942308
EIF2S1	-2.27601	0.980769
EIF2S2	-1.90286	0.980769
EIF2S3	-1.50156	0.961538
EIF3A	-1.51659	0.961538
EIF3B	-1.83742	0.980769
EIF3D	-1.30029	0.884615
EIF3F	-1.33337	0.846154
EIF3G	-1.42612	0.846154
EIF3I	-1.76847	0.961538
EIF4A3	-2.18044	0.980769
EIF4E	-1.51106	0.942308
EIF5	-1.90984	0.980769
EIF6	-1.6659	0.961538
ELOC	-1.1986	0.826923
EPRS1	-1.51578	0.980769
ERH	-2.01916	0.980769
ESF1	-1.17759	0.807692

ESPL1	-1.94188	0.980769
ETF1	-2.27173	0.980769
EXOSC4	-1.40255	0.884615
EXOSC6	-1.62055	0.942308
EXOSC7	-1.15203	0.807692
FARSA	-1.32367	0.826923
FARSB	-1.85391	0.961538
FAU	-2.35276	0.980769
FBL	-1.79887	0.942308
FCF1	-2.07027	0.980769
GARS1	-1.84658	0.980769
GEMIN5	-1.51504	0.942308
GGTLC2	-1.35407	0.903846
GINS1	-2.01828	0.980769
GINS2	-1.82284	0.980769
GINS4	-1.35092	0.846154
GNL2	-1.15682	0.865385
GNL3	-1.34635	0.903846
GPN1	-1.95918	0.980769
GPN2	-1.85576	0.980769
GPN3	-2.10766	0.980769
GPS1	-1.30231	0.846154
GRPEL1	-1.36731	0.961538
GSPT1	-1.79089	0.923077
GTF2A2	-1.50807	0.923077
GTF2B	-1.94251	0.980769
GTF2E1	-1.41547	0.903846
GTF2E2	-1.3277	0.942308
GTF2F2	-1.23176	0.846154
GTF2H1	-1.18606	0.826923
GTPBP4	-1.7519	0.961538
GUK1	-1.29131	0.807692
H2AC15	-1.4015	0.980769
H2AC16	-1.1258	0.865385
H2BC11	-2.06888	0.980769
H2BC15	-1.63988	0.980769
H2BC4	-1.77659	0.980769
H2BC5	-1.36849	0.980769
H2BC6	-1.89887	0.980769
H3C13	-1.82836	0.942308
HARS1	-1.50717	0.903846
HAUS1	-1.62572	0.961538

HAUS5	-1.2884	0.826923
HAUS6	-1.57785	0.942308
HAUS8	-1.33824	0.826923
HCFC1	-1.9647	0.980769
HEATR1	-1.34279	0.923077
HINFP	-1.88149	0.923077
HMGCS1	-1.7575	0.961538
HNRNPC	-1.66985	0.980769
HNRNPK	-2.2822	0.980769
HNRNPL	-1.25292	0.846154
HSPA5	-1.82751	0.980769
HSPA9	-1.84846	0.980769
HSPD1	-1.42462	0.923077
HSPE1	-2.56809	0.980769
IARS1	-1.87712	0.980769
IGBP1	-1.56724	0.961538
IK	-1.65316	0.942308
IMP3	-1.74623	0.961538
INCENP	-1.55126	0.942308
INTS11	-1.92418	0.980769
INTS3	-1.54604	0.961538
INTS4	-1.84249	0.980769
INTS9	-1.80127	0.980769
ISCU	-1.87728	0.980769
ISY1	-1.5354	0.961538
KARS1	-1.86033	0.980769
KAT8	-1.70795	0.980769
KIF11	-2.36071	0.980769
KIF23	-2.07194	0.980769
KIN	-1.61103	0.961538
KPNB1	-2.09328	0.980769
KRR1	-1.45945	0.884615
KRT8	-1.53771	0.961538
LARS1	-1.51241	0.980769
LCE5A	-1.23111	0.980769
LRR1	-2.16277	0.980769
LSM2	-2.11931	0.980769
LSM3	-1.57676	0.980769
LSM4	-1.8932	0.980769
LSM5	-1.34101	0.942308
LSM6	-1.80037	0.980769
LSM7	-1.87567	0.980769

LSM8	-1.70405	0.961538
LTO1	-1.7102	0.980769
LUC7L3	-1.57041	0.980769
MAD2L1	-1.53479	0.942308
MAK16	-1.61165	0.980769
MARS1	-1.57847	0.961538
MASTL	-1.68122	0.923077
MAT2A	-1.46459	0.884615
MCM2	-1.66678	0.942308
MCM4	-1.41262	0.942308
MCM5	-1.3795	0.923077
MCM6	-1.39084	0.903846
MCM7	-1.80186	0.980769
MDN1	-1.642	0.980769
MED11	-1.80224	0.980769
MED14	-1.41735	0.865385
MED20	-1.30722	0.826923
MED22	-1.2577	0.846154
MED28	-1.43376	0.980769
MED30	-1.88359	0.961538
MED31	-1.1723	0.807692
MED4	-1.17464	0.884615
MED6	-1.59099	0.961538
MED8	-1.49126	0.942308
MEPCE	-1.48621	0.961538
MFAP1	-1.62602	0.961538
MMS22L	-1.78417	0.961538
MPHOSPH10	-1.81582	0.961538
MTBP	-2.05015	0.980769
MTREX	-1.64728	0.961538
MYC	-1.65566	0.865385
MZT1	-1.44652	0.961538
NAA50	-1.54419	0.942308
NACA	-1.49481	0.942308
NAPA	-1.86953	0.961538
NARS1	-1.72536	0.942308
NCAPG	-1.50508	0.884615
NCBP1	-1.31667	0.865385
NCBP2	-1.95535	0.980769
NDC80	-1.95287	0.980769
NEDD1	-1.97904	0.980769
NEDD8	-1.7356	0.980769

NFS1	-1.83984	0.961538
NIFK	-1.68833	0.980769
NIP7	-1.73755	0.980769
NLE1	-1.33277	0.923077
NMD3	-1.27922	0.961538
NOL10	-1.41821	0.884615
NOL6	-1.38586	0.865385
NOP16	-1.26108	0.865385
NOP2	-1.31146	0.923077
NOP56	-1.69524	0.980769
NOP58	-1.57631	0.942308
NPLOC4	-1.37835	0.846154
NRF1	-1.56222	0.961538
NSA2	-1.49238	0.961538
NSF	-1.88074	0.980769
NUBP1	-1.29993	0.846154
NUDT21	-2.03879	0.980769
NUF2	-1.7256	0.980769
NUP133	-1.56688	0.961538
NUP160	-1.64245	0.980769
NUP214	-1.60692	0.980769
NUP85	-1.63732	0.961538
NUP93	-1.95676	0.980769
NUS1	-2.14641	0.980769
NUTF2	-2.01565	0.980769
NVL	-1.27759	0.884615
NXF1	-1.58842	0.942308
ORC1	-1.19619	0.826923
ORC6	-1.50505	0.903846
PABPN1	-1.77287	0.961538
PAFAH1B1	-2.33307	0.980769
PAM16	-1.68852	0.980769
PCBP1	-1.35605	0.923077
PCBP2	-1.26035	0.846154
PCNA	-2.42224	0.980769
PDCD11	-1.39733	0.903846
PDRG1	-1.683	0.942308
PES1	-1.26272	0.903846
PFDN2	-2.06205	0.980769
PFDN6	-1.53397	0.942308
PHAX	-1.2838	0.826923
PHB	-2.12518	0.980769

PHB2	-2.2101	0.980769
PHF5A	-2.26873	0.980769
PLK1	-2.33207	0.980769
PMPCB	-1.52688	0.942308
POLA2	-1.45702	0.980769
POLD1	-1.79642	0.980769
POLD2	-1.43922	0.923077
POLD3	-1.72912	0.980769
POLE	-1.95719	0.980769
POLE2	-1.67584	0.961538
POLR1A	-1.82345	0.980769
POLR1B	-1.36785	0.903846
POLR1C	-1.89885	0.980769
POLR1F	-1.45989	0.923077
POLR2B	-2.11221	0.980769
POLR2C	-2.09799	0.980769
POLR2D	-1.89039	0.980769
POLR2E	-1.91854	0.980769
POLR2F	-1.8716	0.980769
POLR2G	-1.72189	0.980769
POLR2H	-1.6552	0.980769
POLR2I	-1.95257	0.980769
POLR2L	-2.59118	0.980769
POLR3A	-2.05788	0.980769
POLR3B	-1.81573	0.980769
POLR3C	-1.65786	0.942308
POLR3F	-1.65374	0.961538
POLR3H	-1.40747	0.884615
POLR3K	-1.81663	0.980769
POP5	-1.51261	0.884615
PPAN	-1.24667	0.923077
PPIL2	-1.3315	0.865385
PPP1R11	-1.25884	0.846154
PPWD1	-1.56941	0.903846
PRC1	-1.67263	0.961538
PRELID1	-2.05928	0.961538
PRELID3B	-1.77381	0.980769
PRIM1	-1.94701	0.980769
PRPF19	-2.18382	0.980769
PRPF31	-2.01915	0.980769
PRPF38A	-2.36189	0.980769
PRPF38B	-2.09006	0.980769

PRPF4	-1.12096	0.826923
PRPF6	-1.62899	0.961538
PRPF8	-2.13537	0.980769
PSMA1	-2.27358	0.980769
PSMA2	-2.0531	0.980769
PSMA3	-2.33093	0.980769
PSMA4	-1.85194	0.980769
PSMA5	-2.11311	0.980769
PSMA6	-2.38142	0.980769
PSMA7	-2.0471	0.980769
PSMB1	-1.73308	0.980769
PSMB2	-1.92789	0.980769
PSMB3	-2.34923	0.980769
PSMB4	-2.19368	0.980769
PSMB5	-1.72116	0.846154
PSMB7	-1.52874	0.961538
PSMC1	-1.23536	0.865385
PSMC2	-1.36679	0.923077
PSMC3	-1.72461	0.980769
PSMC4	-1.53344	0.980769
PSMC5	-1.40897	0.961538
PSMC6	-1.92326	0.980769
PSMD1	-1.4163	0.807692
PSMD11	-1.67715	0.980769
PSMD12	-1.52376	0.961538
PSMD14	-1.7056	0.961538
PSMD2	-1.37861	0.884615
PSMD3	-1.73451	0.980769
PSMD4	-1.53964	0.942308
PSMD6	-1.80133	0.980769
PSMD7	-1.60473	0.961538
PSMD8	-1.37638	0.980769
PSMG3	-1.6005	0.865385
PUF60	-2.33159	0.980769
PWP2	-1.44172	0.923077
QARS1	-1.69742	0.961538
RABGGTA	-1.55937	0.923077
RABGGTB	-1.43833	0.865385
RACGAP1	-1.37671	0.923077
RACK1	-1.58941	0.923077
RAD21	-2.09415	0.980769
RAD51	-1.31363	0.865385

RAN	-2.76253	0.980769
RANGAP1	-1.78409	0.980769
RBBP4	-1.31108	0.884615
RBM14	-1.26506	0.826923
RBM17	-1.5123	0.903846
RBM22	-1.99115	0.980769
RBM25	-1.52888	0.923077
RBM39	-1.9208	0.980769
RBM8A	-1.62639	0.942308
RBMX	-1.35228	0.903846
RBX1	-1.36968	0.961538
RCC1	-1.41862	0.903846
RFC2	-1.54488	0.923077
RFC3	-1.48315	0.865385
RFC5	-1.46632	0.942308
RIOK2	-1.36556	0.903846
RNGTT	-2.20112	0.961538
RNPC3	-1.7527	0.980769
RNPS1	-1.31135	0.826923
RPA1	-2.24732	0.980769
RPA2	-1.8983	0.980769
RPA3	-1.8572	0.980769
RPAIN	-1.38972	0.807692
RPAP1	-1.99328	0.980769
RPAP2	-1.52886	0.980769
RPL10A	-2.0743	0.980769
RPL11	-2.30504	0.980769
RPL12	-2.13857	0.980769
RPL13	-2.1722	0.980769
RPL13A	-1.91653	0.980769
RPL14	-1.91467	0.980769
RPL15	-2.56442	0.980769
RPL18	-1.95761	0.980769
RPL18A	-2.16919	0.980769
RPL19	-2.03892	0.980769
RPL21	-2.20332	0.980769
RPL23	-2.3882	0.980769
RPL23A	-1.85084	0.980769
RPL24	-1.83612	0.961538
RPL26	-1.80129	0.980769
RPL27	-2.10751	0.980769
RPL27A	-1.92181	0.980769

RPL3	-2.03413	0.980769
RPL30	-1.43388	0.961538
RPL31	-2.33382	0.980769
RPL32	-2.05449	0.980769
RPL35	-1.68105	0.980769
RPL35A	-1.24883	0.884615
RPL36	-1.67544	0.980769
RPL37	-1.41555	0.961538
RPL37A	-1.87628	0.980769
RPL38	-1.27071	0.865385
RPL4	-2.51111	0.980769
RPL5	-2.26312	0.980769
RPL6	-1.9195	0.961538
RPL7	-1.77243	0.980769
RPL8	-2.35736	0.980769
RPLP0	-2.10583	0.980769
RPLP1	-1.7198	0.980769
RPLP2	-1.49175	0.865385
RPP30	-1.33088	0.884615
RPS10	-1.42863	0.980769
RPS11	-2.24587	0.980769
RPS12	-2.19603	0.961538
RPS13	-2.37836	0.980769
RPS15	-2.02015	0.980769
RPS15A	-2.10702	0.980769
RPS16	-1.92475	0.961538
RPS18	-2.20741	0.980769
RPS19	-2.38565	0.980769
RPS2	-2.17138	0.980769
RPS20	-2.40419	0.980769
RPS21	-1.4507	0.980769
RPS23	-1.84905	0.980769
RPS24	-1.82779	0.980769
RPS25	-1.36449	0.884615
RPS27A	-2.30778	0.980769
RPS29	-2.41159	0.980769
RPS3	-2.2015	0.980769
RPS4X	-2.13577	0.923077
RPS5	-1.99705	0.980769
RPS6	-2.44796	0.980769
RPS7	-2.04016	0.980769
RPS8	-2.35237	0.980769

RPS9	-2.20048	0.980769
RPSA	-2.04342	0.980769
RRM1	-2.45414	0.980769
RRM2	-2.52048	0.980769
RRN3	-2.01391	0.980769
RRP12	-1.31049	0.884615
RSL1D1	-1.33676	0.923077
RUVBL1	-1.86762	0.980769
RUVBL2	-1.74968	0.980769
SACM1L	-1.35931	0.923077
SAP18	-1.57683	0.942308
SAP30BP	-1.94418	0.980769
SARS1	-2.35983	0.980769
SART3	-1.7388	0.980769
SBDS	-1.54137	0.865385
SBNO1	-1.62586	0.980769
SCFD1	-1.65573	0.942308
SDAD1	-1.28251	0.846154
SDE2	-2.05708	0.961538
SEC13	-1.30985	0.923077
SEC61A1	-1.92299	0.980769
SEC61G	-1.516	0.980769
SF1	-2.13529	0.980769
SF3A1	-2.19472	0.980769
SF3A2	-1.80651	0.980769
SF3A3	-2.01171	0.980769
SF3B1	-1.6751	0.903846
SF3B2	-1.28914	0.807692
SF3B3	-1.99418	0.980769
SF3B4	-1.79085	0.980769
SF3B5	-2.34804	0.980769
SF3B6	-1.69274	0.980769
SFPQ	-2.04301	0.980769
SKP1	-1.37229	0.923077
SLC39A7	-1.6335	0.846154
SLU7	-1.52378	0.942308
SMC1A	-1.55575	0.942308
SMC2	-1.48981	0.980769
SMC4	-1.82602	0.961538
SMG1	-1.42292	0.923077
SMR3B	-1.56779	0.980769
SMU1	-2.24241	0.980769

SNAPC1	-1.62873	0.942308
SNAPC2	-1.61206	0.826923
SNAPC4	-1.72506	0.961538
SNAPC5	-1.43148	0.807692
SNRNP200	-2.38707	0.961538
SNRNP25	-1.47073	0.961538
SNRNP27	-1.31044	0.903846
SNRNP35	-1.61424	0.980769
SNRNP70	-1.44633	0.961538
SNRPA1	-2.38964	0.980769
SNRPB	-2.46858	0.980769
SNRPC	-1.12508	0.807692
SNRPD1	-2.20659	0.980769
SNRPD2	-2.20904	0.980769
SNRPF	-2.42035	0.980769
SNU13	-2.27255	0.980769
SNW1	-1.63276	0.961538
SPC24	-2.08867	0.961538
SPC25	-1.60405	0.980769
SPCS2	-1.36719	0.884615
SPDL1	-1.29656	0.961538
SPOUT1	-1.32822	0.903846
SRBD1	-1.44294	0.942308
SRP19	-1.17986	0.884615
SRP54	-1.91155	0.961538
SRP72	-1.41528	0.942308
SRP9	-1.69929	0.980769
SRSF1	-1.66839	0.980769
SRSF2	-2.07431	0.980769
SRSF3	-2.15744	0.980769
SRSF7	-2.24911	0.980769
SS18L2	-1.84646	0.923077
SSRP1	-1.48152	0.980769
SSU72	-1.90661	0.961538
STX5	-1.60522	0.961538
SUPT16H	-1.85335	0.980769
SUPT5H	-1.50694	0.826923
SUPT6H	-1.98286	0.980769
SYMPK	-1.3557	0.884615
TAF6	-1.79656	0.961538
TANGO6	-1.37656	0.903846
TARS1	-2.0181	0.980769

TCP1	-1.73667	0.980769
THOC2	-2.10155	0.980769
THOC3	-1.79378	0.961538
THOC5	-1.22333	0.826923
THOC7	-1.18034	0.865385
TICRR	-1.46102	0.942308
TIGD1	-1.15866	0.826923
TIMELESS	-1.89591	0.980769
TIPIN	-1.16516	0.807692
TNPO3	-1.67326	0.980769
TOMM40	-1.38721	0.865385
TONSL	-1.74681	0.980769
TOP2A	-2.01078	0.980769
TOPBP1	-1.49332	0.961538
TRAPPC3	-1.49994	0.923077
TRAPPC4	-1.1689	0.807692
TRAPPC8	-1.42408	0.923077
TRMT112	-1.8097	0.961538
TRRAP	-1.60783	0.923077
TSR1	-1.24039	0.846154
TSR2	-1.9938	0.961538
TTC27	-1.41509	0.961538
TUBGCP2	-1.26849	0.884615
TUBGCP3	-1.32015	0.923077
TUT1	-2.04054	0.980769
TXNL4A	-2.42445	0.980769
U2AF2	-2.04689	0.980769
U2SURP	-1.56551	0.961538
UBA1	-2.14512	0.980769
UBA2	-1.26422	0.846154
UBA52	-1.66282	0.961538
UBE2I	-1.67676	0.961538
UBL5	-2.4563	0.980769
UBTF	-1.53575	0.980769
UFD1	-1.36377	0.903846
UPF1	-1.32295	0.903846
UPF2	-1.33828	0.923077
URI1	-1.71339	0.961538
USP36	-1.33667	0.942308
USP39	-1.41203	0.923077
USP5	-1.75562	0.980769
USPL1	-1.33003	0.865385

UTP15	-1.66749	0.980769
UTP20	-1.50892	0.961538
UTP4	-1.53622	0.961538
VARS1	-1.62633	0.980769
VCP	-2.22301	0.980769
VIRMA	-1.31187	0.884615
VPS25	-1.88617	0.961538
VPS28	-2.06337	0.980769
WARS1	-1.52195	0.961538
WDR12	-1.42387	0.961538
WDR3	-1.14102	0.826923
WDR33	-1.41531	0.980769
WDR43	-2.02574	0.980769
WDR5	-1.55772	0.903846
WDR70	-1.97986	0.980769
WDR74	-1.94628	0.980769
WDR75	-1.3384	0.826923
WDR77	-1.43932	0.865385
WDR82	-1.62001	0.923077
WEE1	-2.22991	0.980769
XAB2	-2.01258	0.980769
XPO1	-1.81144	0.980769
XRCC5	-1.15832	0.826923
XRCC6	-1.70211	0.980769
YARS1	-1.9064	0.980769
YJU2	-1.91094	0.980769
YKT6	-1.60609	0.942308
YRDC	-1.69513	0.865385
ZMAT2	-1.59384	0.961538
ZMAT5	-1.30586	0.884615
ZNF131	-1.25785	0.807692
ZNF207	-1.97286	0.980769
ZNF830	-1.4043	0.884615
ZNHIT2	-1.86971	0.980769
ZPR1	-1.45713	0.923077
